# Validation of the Lithuanian version of the Eating Disorder Examination Questionnaire 6.0 in a student sample

**DOI:** 10.1002/brb3.1555

**Published:** 2020-01-28

**Authors:** Migle Baceviciene, Vaiva Balciuniene, Rasa Jankauskiene

**Affiliations:** ^1^ Department of Physical and Social Education Lithuanian Sports University Kaunas Lithuania; ^2^ Institute of Sport Science and Innovation Lithuanian Sports University Kaunas Lithuania

**Keywords:** Eating Disorder Examination Questionnaire 6.0, factor structure, Lithuanian translation, reliability, students, validity

## Abstract

**Background and Objectives:**

The Eating Disorder Examination Questionnaire 6.0 (EDE‐Q 6.0) is one of the most broadly used self‐report tools that assesses attitudes and behaviors associated with eating disorders (EDs). The aim of the present study was to examine the reliability, validity, and factor structure of the Lithuanian version of the EDE‐Q 6.0 (LT‐EDE‐Q 6.0) in a nonclinical student sample.

**Materials and Methods:**

A sample of 382 students (mean age 24.0 ± 6.4) participated in the study. The students completed a self‐report questionnaire measuring the risk of EDs (LT‐EDE‐Q 6.0), body image (LT‐MBSRQ‐AS), quality of life (LT‐WHOQOL‐BREF), and self‐esteem (RSES). Cronbach's alpha assessed the internal consistency of the EDE‐Q 6.0. Pearson's correlations were used for the analyses of the construct and concurrent validity with the subscales of LT‐MBSRQ‐AS, LT‐WHOQOL‐BREF, and RSES. Intraclass correlation coefficients (ICC) were calculated for assessing test‐retest reliability.

**Results:**

The mean score of the LT‐EDE‐Q 6.0 in the mixed sample was 1.5 ± 1.02. For women and men, the general mean scores were higher than in the majority of the samples of Western Europe but lower than in the United States. Acceptable internal consistency for the four subscales (0.75–0.88) and the LT‐EDE‐Q 6.0 general score (0.94) was obtained. Test‐retest reliability was good to excellent for all subscales (0.66–0.91) and for the items that assessed essential behavioral features of EDs (0.84–0.90, except item 14 ICC = 0.4). The LT‐EDE‐Q 6.0 scores had adequate concurrent validity. However, the original 4‐factor structure or other proposed models of EDE‐Q were not obtained by CFA.

**Conclusions:**

The results of the current study support the applicability, validity, and reliability of the LT‐EDE‐Q 6.0 in a nonclinical Lithuanian student sample. However, we recommend assessing the general scale score without the application of the subscales. The Lithuanian version of this instrument should be further investigated with clinical samples to identify clinically diagnosed cases.

## Graphical abstract

The Eating Disorder Examination Questionnaire 6.0 (EDE‐Q 6.0) is one of the most broadly used self‐report tools that assesses attitudes and behaviors associated with eating disorders (EDs). The aim of the present study was to examine the reliability, validity, and factor structure of the Lithuanian version of the EDE‐Q 6.0 (LT‐EDE‐Q 6.0) in a nonclinical student sample. The results of the current study preliminarily support the applicability, validity, and reliability of the LT‐EDE‐Q 6.0 in a nonclinical Lithuanian student sample. The Lithuanian version of this instrument should be further investigated with clinical samples to identify clinically diagnosed cases.

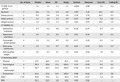

## 1 INTRODUCTION

Body image concerns and disordered eating are major health problems in youth (Neumark‐Sztainer et al., 2018). The most reliable method for diagnosing and assessing eating disorders (EDs) is a structured or semistructured clinical interview administered by trained clinicians (Fairburn & Beglin, 1994). However, researchers and clinicians need an alternative method for screening clinical and nonclinical individuals in various population groups. Self‐report questionnaires are more time/cost‐effective and do not require specialized training in the field of ED epidemiological studies and for assessing the effect of preventive attempts and treatments (Ciao, Loth, & Neumark‐Sztainer, 2014; Cooper & Fairburn, 1987; Fairburn & Beglin, 1994).

The Eating Disorder Examination Questionnaire 6.0 (EDE‐Q 6.0) is one of the most broadly used self‐report methods that assesses behaviors and attitudes associated with EDs (Fairburn & Beglin, 1994, 2008), and it can be applied when it is impossible or unacceptable to conduct an interview assessment (Berg, Peterson, Frazier, & Crow, 2012; Fairburn & Beglin, 1994). The tool has been derived from the full‐length semistructured interview‐based Eating Disorder Examination (EDE), which has been considered the gold standard in the assessment of the specific psychopathology of eating‐disordered behavior (Cooper & Fairburn, 1987; Fairburn & Cooper, 1993; Fairburn, Cooper, & O'Connor, 2008). Psychometric characteristics of the EDE and EDE‐Q were considered consistent, as reviewed in a meta‐analysis (Berg et al., 2012). It has been concluded that both instruments are capable of measuring ED psychopathology in various epidemiologic and clinical study populations, including individuals with an ED diagnosis (Berg et al., 2012).

The original version of the EDE‐Q 6.0 has been extensively studied, and its good psychometric properties have been globally demonstrated in Portuguese, Spanish, Japanese, Hebrew, Persian, Mexican, and others samples (Machado et al., 2014; Mahmoodi, Moloodi, & Ghaderi, 2016; Mitsui, Yoshida, & Komaki, 2017; Peláez‐Fernández, Labrador, & Raich, 2012; Unikel Santoncini et al., 2018; Zohar, Lev‐Ari, & Bachner‐Melman, 2017). In terms of reliability, some researchers have shown that the EDE‐Q 6.0 has good to strong internal consistency (Calugi et al., 2017; Giovazolias, Tsaousis, & Vallianatou, 2013; Mahmoodi et al., 2016; Yucel et al., 2011), test‐retest reliability (Calugi et al., 2017; Yucel et al., 2011), an adequate convergent validity (Giovazolias et al., 2013; Mahmoodi et al., 2016; Peláez‐Fernández et al., 2012; Zohar et al., 2017), divergent validity (Giovazolias et al., 2013; Mahmoodi et al., 2016; Zohar et al., 2017), and criterion‐oriented validity (Calugi et al., 2017; Yucel et al., 2011). Several studies have verified the sensitivity and specificity of the EDE‐Q 6.0 (Peláez‐Fernández et al., 2012).

The original and theorized 22‐item, 4‐factor structure of the scale has been divided into four subscales: restraint, eating concern, shape concern, and weight concern (Fairburn & Beglin, 2008). However, rivaling structures have been found in other studies with the EDE‐Q. A Hebrew study using EFA and CFA analyses with 292 community volunteers (18% were male) principally confirmed the original factor structure; nevertheless, weight and shape concerns merged into one factor (Zohar et al., 2017). Grilo, Reas, Hopwood, and Crosby, (2015) analyzed the responses of a nonclinical sample of male and female students in the USA and based on CFA supported a modified 7‐item 3‐factor structure, where the three factors were designated dietary restraint, shape and weight overvaluation, and body dissatisfaction. Notably, an abbreviated and modified 7‐item 3‐factor version has received research support in a nonclinical sample in Portugal of female high school and college student and treatment‐seeking patients with ED diagnoses (Machado, Grilo, & Crosby, 2018), Mexican female students and ED patients (Unikel Santoncini et al., 2018) and Canadian university students and a middle‐aged American community sample (Tobin, Lacroix, & von Ranson, 2019). Giovazolias et al. (2013) used CFA to investigate the latent structure of the Greek EDE‐Q in a sample of 500 university female students and found that the Swedish 22‐item, 3‐factor solution proposed by Peterson et al. (2007) had a better fit than the theorized 22‐item, 4‐factor model (Fairburn & Beglin, 1994), the German Hilbert's 17‐item, 3‐factor model (Hilbert, Tuschen‐Caffier, Karwautz, Niederhofer, & Munsch, 2007), and the 1‐factor model, which assumes that a single latent factor underlies all the EDE‐Q items (Byrne, Allen, Lampard, Dove, & Fursland, 2010; Wade, Byrne, & Bryant‐Waugh, 2008). Moreover, Gideon et al. (2016) followed 489 individuals aged 18–72 with various EDs recruited from three UK specialist eating disorder services using the EDE‐Q 6.0 and developed and validated a 12‐item short form of the EDE‐Q 6.0 (EDE‐QS).

To the best of our knowledge, there are no reliable and valid instruments for ED screening in various age groups in Lithuania. There is some evidence that the Eating Disorder Inventory‐2 (EDI‐2; Garner, 1991) was translated and validated in Lithuania. However, the results were presented in a doctoral dissertation two decades ago and have not been published internationally (Aputytė, 2000). To date, no epidemiological studies on the prevalence of eating disorders or disordered eating were performed in Lithuania. However, body image concerns, health‐compromising eating behaviors, disordered eating, and the prevalence of psychosomatic and psychiatric disorders constitute a significant problem and area of research in young people globally and in Lithuania (Baceviciene, Jankauskiene, & Emeljanovas, 2019; Jankauskiene & Baceviciene, 2019; Lesinskiene et al., 2018). The reduction in EDs and health‐compromising eating behaviors is one of the most important targets in prevention programs for obesity and body image concerns (Ciao et al., 2014). Therefore, it is crucial to have reliable measures to evaluate the effect of interventions in Lithuania. Self‐report questionnaires, as tools that do not require specialized training, are needed for evaluating the outcomes of preventive efforts and treatment in education/prevention programs (Ciao et al., 2014; Cooper & Fairburn, 1987; Fairburn & Beglin, 1994). Thus, the present study aimed to examine the reliability, validity, and factor structure of the Lithuanian version of the EDE‐Q 6.0 (LT‐EDE‐Q 6.0) as a screening self‐report questionnaire for EDs in a nonclinical Lithuanian student sample.

## 2 MATERIALS AND METHODS

### 2.1 Participants

A mixed‐gender sample of undergraduate (*n* = 298) and graduate students (*n* = 84) from various state universities and colleges located in Lithuania participated in this study. The sample consisted of 382 students (95 were males). The mean age of the sample was 24.0 ± 6.4 years. The majority of the sample was in the 18–30 age range (*n* = 365, 95.6%). About 295 (77.2%) of participants studied in universities, while 70 (22.8%) were students in colleges.

### 2.2 Procedure

The data were obtained in Lithuanian state universities and colleges during April‐June in 2019. The present study is a part of a more extensive study in which the representativeness of the sample of students was achieved by the compliance of the respondents to the numbers of students in all study areas. Thus, accordingly to the distribution of the general numbers, students in this sample were enrolled in natural and agricultural (2.6%), technology (10.6%), medical and health (24.9%), social and humanities (61.9%) study areas. The researcher V.B. collected the data contacting the administration of the universities and colleges. After having word consent from administrative staff, questionnaires for students were provided. The sample of students was from seven universities and two colleges (out of thirteen state universities and twelve state colleges in Lithuania). The procedure was scheduled in‐class time, with no time limit, yet the approximate time for filling the questionnaires was 45 min. To increase the motivation of the students to complete the survey fully, an emotional, motivational incentive to enroll in the study was created. Students were informed that completing the questionnaire fully and answering all questions honestly, will open them an opportunity to remotely listen to a free four‐hour webinar “Healthy Nutrition and Weight Control”. Three hundred and ninety‐three questionnaires were completed; no students refused to participate in the study. However, eleven questionnaires were excluded from the study if not all items in the survey were appropriately completed (not appropriate answers were provided). Therefore, three hundred and eighty‐two questionnaires were used in the present study.

### 2.3 Ethical considerations

The researchers received ethical approval to conduct this study by the Committee for Social Sciences Research Ethics of the Lithuanian Sports University (protocol No. SMTEK‐7, 13‐03‐2019). Following the fundamental ethical and legal principles of the research, the students were introduced to the purpose of the study before the questionnaires were presented. The laws of anonymity, goodwill, and volunteering were followed during the survey. To avoid violating national and EU legislation, the students were instructed to mark the response “I agree to participate” or “I disagree to participate” to give their consent to participate in the study before beginning the survey.

### 2.4 Measures

#### 2.4.1 Demographic data

Participants in the study were asked to specify their gender, age, type of the higher education institution (university or college), the level of study cycle, study area, study program, and the year of study.

#### 2.4.2 Body mass index

Body mass index (BMI) was based on the self‐reported data of the students' height, and weight from which BMI was calculated (kg/m^2^). For sample characteristics, as recommended by the World Health Organization classification, the students' BMI was classified into four body mass categories: underweight (BMI < 18.5 kg/m^2^), normal weight (BMI = 18.5–24.9 kg/m^2^), overweight (BMI = 25.0–29.9 kg/m^2^), and obese (BMI ≥ 30.0 kg/m^2^; World Health Organization, 1997). The BMI ranged from 15.8 to 36.2 (*M* = 22.9, *SD* = 3.9) kg/m^2^. The majority of the sample (*n* = 261, 68.3%) was of normal weight, 87 (22.7%) were overweight or obese, 34 (8.9%) were underweight.

#### 2.4.3 The Eating Disorder Examination Questionnaire 6.0

The Eating Disorder Examination Questionnaire 6.0 (EDE‐Q 6.0; Fairburn & Beglin, 2008) is a 28‐item self‐report questionnaire and provides a comprehensive evaluation of the essential behavioral characteristics of EDs and eating‐disordered behavior. It was obtained from the official site (https://www.cbte.co/for-professionals/measures/) where it was stated that the questionnaire is freely available only for noncommercial research use, and no permission needs to be queried.

The translation of the EDE‐Q 6.0 into Lithuanian was carefully performed by two professional translators and then back‐translated to English by two professional translators from a translation agency in Kaunas, Lithuania. The final translation was reviewed by an expert in the field of EDs to determine whether the questionnaire covered the concepts it aims to measure. The face validity was rated as good.

The EDE‐Q 6.0 concentrates on the last 28 days and establishes two models of data. First, the six open‐ended questions (from 13 to 18) result in frequency data on the essential behavioral characteristics of EDs (number of episodes of the behavior or number of days on which the action has occurred): objective binge eating, self‐induced vomiting, laxative use, and excessive exercise. Second, 22 attitudinal questions comprise four subscales and result in subscale scores that reflect the severity of the ED characteristics. The restraint subscale composed of five items (1, 2, 3, 4, and 5) indicates the restriction of eating behavior. The 5‐item (7, 9, 19, 20, and 21) eating concern subscale reveals anxiety and fears about eating. The 8‐item (6, 8, 10, 11, 23, 26, 27, and 28) shape concern subscale evaluates anxiety and concern about body forms. The 5‐item (8, 12, 22, 24, and 25) weight concern subscale measures fears and anxiety about body weight. The answer options are arranged on a 7‐point Likert scale from 0 (no day) to 6 (every day). A higher score reflects either greater severity or frequency.

#### 2.4.4 The Lithuanian version of the Multidimensional Body‐Self Relations Questionnaire–Appearance Scales

The Lithuanian version of the Multidimensional Body‐Self Relations Questionnaire–Appearance Scales (MBSRQ‐AS; Brown, Cash, & Mikulka, 1990) was employed to assess the appearance‐related elements of the body image construct. This instrument of 34 items consists of five subscales, with responses on a 5‐point Likert scale ranging from 1 (completely disagree) to 5 (completely agree). The 7‐item (3, 5, 9, 12, 15, 18, and 19) appearance evaluation subscale determines perceptions of physical attractiveness, with a higher score reflecting a higher appearance evaluation. The appearance orientation subscale consists of 12 items (1, 2, 6, 7, 10, 11, 13, 14, 16, 17, 20, and 21) and reveals the degree of investment in one's appearance, with a higher score indicating a higher appearance orientation. The body area satisfaction subscale consists of nine items (from 26 to 34). It evaluates satisfaction or dissatisfaction with particular areas of the body on a 5‐point Likert scale ranging from 1 (very dissatisfied) to 5 (very satisfied). A higher score defines greater body area satisfaction. The 4‐item (4, 8, 22, and 23) overweight preoccupation subscale evaluates weight vigilance, dieting, fat anxiety, and eating restraint. A higher score defines a greater preoccupation with being overweight. The 2‐item (24 and 25) self‐classified weight scale shows how one perceives and identifies one's weight on a 5‐point Likert scale ranging from 1 (very underweight) to 5 (very overweight). A higher score indicates firmer beliefs that bodyweight is too high. Research has supported the reliability and validity of the Lithuanian version of the MBSRQ‐AS (LT‐MBSRQ‐AS) in a student population sample (Miškinytė & Bagdonas, 2010). The scale was derived from the official site (http://www.body-images.com) with the purchased authorization of the author Thomas F. Cash, Ph.D. The internal consistency of the subscales appearance orientation, appearance evaluation, body area satisfaction, overweight preoccupation, and self‐classified weight was 0.83, 0.78, 0.86, 0.71, and 0.82, respectively. Cronbach's alpha for the LT‐MBSRQ‐AS general scale was 0.75.

#### 2.4.5 The Lithuanian version of the World Health Organization Quality of Life‐BREF Questionnaire

The Lithuanian version of The World Health Organization Quality of Life‐BREF Questionnaire (WHOQOL‐BREF; World Health Organization, 1998a) is an abbreviated version of the World Health Organization Quality of Life‐100 (WHOQOL‐100; World Health Organization, 1998b) self‐report questionnaire with 26 items and was used to assess the quality of life. Two questions of the overall perception of the quality of life and the overall understanding of health were evaluated separately. The remaining 24 items of the questionnaire comprise four domains. The 7‐item (3, 4, 10, 15, 16, 17, and 18) physical health domain includes questions about dependence on medicinal substances and medical aids, pain and discomfort, activities of daily living, energy and fatigue, mobility, sleep and rest, and work capacity. The 6‐item (5, 6, 7, 11, 19, and 26) psychological health domain includes questions about self‐esteem, body image and appearance, negative and positive feelings, spirituality‐religion, personal beliefs, and concentration. The social relations domain assesses personal relationships, social support, and sexual activity and consists of three items (from 20 to 22). The 8‐item (8, 9, 12, 13, 14, 23, 24, and 25) environment domain reveals information about one's financial resources, physical safety, home environment, the possibility for recreation, opportunities for obtaining new skills and knowledge, health and social care, physical environment, and transportation satisfaction. The responses can range from 1 (very dissatisfied) to 5 (very satisfied). The scores are transformed into a scale between 0 and 100, with 0 being very poor and 100 being very good. The reliability and validity of the Lithuanian version of the WHOQOL‐BREF (LT‐WHOQOL‐BREF) in a student population sample have been demonstrated (Dučinskienė, Kalėdinė, Petrauskienė, & Šumskas, 2002). The questionnaire was obtained from the official site of the World Health Organization (http://depts.washington.edu/seaqol/WHOQOL-BREF). The internal consistency of the domains of physical health, psychological health, social relations, and the environment was 0.75, 0.83, 0.74, and 0.83, respectively. Cronbach's alpha for the LT‐WHOQOL‐BREF general scale was 0.92.

#### 2.4.6 The Lithuanian version of M. Rosenberg's Self‐Esteem Scale

The Lithuanian version of M. Rosenberg's Self‐Esteem Scale (RSES; Rosenberg, 1979) was used to assess self‐esteem and general feelings of self‐worth. The scale is composed of 10 items scored on a 4‐point Likert scale ranging from 1 (strongly disagree) to 4 (strongly agree), yielding scores from 10 to 40. After reversing the positively worded items, an overall self‐esteem score is computed. A higher score denotes a greater level of self‐esteem. RSES is the most widely used measure of global self‐esteem (Schmitt & Allik, 2005). The instrument may be used without explicit permission. Cronbach's alpha for the RSES in this study was 0.91.

### 2.5 Statistical analysis

First, descriptive statistics of the sample were performed, and the results are presented as the means ± standard deviations and as percentages according to the type of variable. Normative data for the LT‐EDE‐Q 6.0 were presented using descriptive statistics. Second, Cronbach's alpha coefficients were used for the evaluation of internal consistency. A score of ≥0.90 was considered excellent, ≥0.80 good, and ≥0.70 acceptable. Thirty students were selected to complete the same questionnaire two weeks after they first had completed surveys to investigate the test‐retest reliability of the LT‐EDE‐Q 6.0. Intraclass correlation coefficients (ICCs) were calculated for assessing test‐retest reliability. Pearson's correlation coefficients were used for the analyses of construct validity (interitem correlations and divergent validity). Third, to confirm the concurrent validity, Pearson's correlation coefficients were used to evaluate the relationships between the subscale scores from the LT‐EDE‐Q 6.0 and the measures from the LT‐MBSRQ‐AS, LT‐WHOQOL‐BREF, RSES, and BMI calculation. The statistical analyses were carried out using IBM SPSS Statistics 25 (IBM Corp.). Finally, using AMOS version 24, confirmatory factor analysis (CFA) of the 22‐item scale of the LT‐EDE‐Q 6.0 was carried out to check the agreement of its factorial structure with the theorized version. The goodness of fit of the model was assessed using acceptable fit values: the *χ* ^2^/*df* (2 < *χ* ^2^/*df* < 3); the goodness of fit index, GFI (0.90 < GFI < 0.95); the comparative fit index, CFI (0.90 < CFI < 0.95); the adjusted goodness of fit index, AGFI (0.85 < AGFI < 0.90); and the root of the mean square error of approximation, RMSEA (0.05 < RMSEA < 0.08).

## 3 RESULTS

The descriptive statistics for the LT‐EDE‐Q 6.0, LT‐MBSRQ‐AS, LT‐WHOQOL‐BREF, RSES, and BMI results are presented in Table 1. The number of items in the scale, median, mean, standard deviation, range, kurtosis, skewness, and percent scoring at the lowest possible value (floor) and the highest possible value (ceiling) was presented to report the statistical characteristics of the study scales. The general score of LT‐EDE‐Q 6.0 was 1.5 ± 1.2. For women, a general score of 1.64 ± 1.22 was higher compared with men 1.08 ± 1.07 (*p* < .001). The mean scores for the LT‐EDE‐Q 6.0 subscales ranged from 0.8 ± 1.0 (eating concern subscale) to 2.0 ± 1.5 (shape concern subscale). The LT‐EDE‐Q 6.0 subscales were found to be positively skewed. The skewness and kurtosis coefficients were computed for univariate normality analysis purposes, and all values were within ±1, except for the restraint subscale (skewness was 1.26) and eating concern subscale (skewness was 1.74, kurtosis was 2.78). The floor effects for the LT‐EDE‐Q 6.0 ranged from 2.6% (LT‐EDE‐Q 6.0 general) to 26.7% (eating concern subscale), and the ceiling effects ranged from 0.3% (eating concern subscale/LT‐EDE‐Q 6.0 general) to 1.0% (weight concern subscale).

**Table 1:** Descriptive statistics of the study scales

	No. of items	Median	Mean	*SD*	Range	Kurtosis	Skewness	Floor (%)	Ceiling (%)
LT‐EDE‐Q 6.0 general		1.2	1.5	1.2	5.4	−0.004	0.88	2.6	0.3
Restraint	5	0.8	1.2	1.4	6.0	0.89	1.26	25.1	0.5
Eating concern	5	0.4	0.8	1.0	4.8	2.78	1.74	26.7	0.3
Shape concern	8	1.6	2.0	1.5	6.0	−0.41	0.69	6.5	0.8
Weight concern	5	1.0	1.5	1.5	6.0	0.01	0.93	18.6	1.0
LT‐MBSRQ‐AS									
Appearance evaluation	7	3.3	3.2	0.8	4.0	−0.36	−0.19	0.3	1.0
Appearance orientation	12	3.4	3.5	0.6	3.3	0.41	−0.24	0.5	0.3
Overweight preoccupation	4	2.3	2.3	0.9	4.0	−0.27	0.52	0.5	1.6
Body areas satisfaction	9	3.2	3.2	0.7	4.0	0.01	−0.10	10.5	0.3
Self‐classified weight	2	3.0	3.2	0.7	4.0	0.65	0.06	1.0	3.4
LT‐WHOQOL‐BREF									
Physical	7	67.9	68.0	15.5	82.1	0.05	−0.49	0.3	0.5
Psychological	6	58.3	58.8	17.6	100.0	0.19	−0.43	0.3	0.5
Social relationships	3	66.7	60.5	23.0	100.0	−0.50	−0.33	0.8	6.0
Environment	8	65.6	65.6	16.9	100.0	0.88	−0.66	0.3	0.8
RSES	1–10	30.0	29.6	6.1	30.0	0.70	−0.57	1.3	5.0
BMI	—	22.3	22.9	3.8	20.4	0.82	0.96	—	—

Abbreviations: BMI, body mass index; LT‐EDE‐Q 6.0, Lithuanian version of the Eating Disorder Examination Questionnaire 6.0; LT‐MBSRQ‐AS, Lithuanian version of the Multidimensional Body‐Self Relations Questionnaire–Appearance Scales; LT‐WHOQOL‐BREF, Lithuanian version of the World Health Organization Quality of Life‐BREF Questionnaire; RSES, M. Rosenberg Self‐Esteem Scale; *SD*, standard deviation.

Table 2 presents the essential behavioral features of EDs and shows the proportion of students who engaged in any or the regular occurrence of disordered eating behaviors (dietary restraint, binge eating distinguished by loss of control) and compensatory behaviors (self‐induced vomiting, use of laxatives, and excessive exercising) during the preceding 28 days.

**Table 2:** The proportion of students who engaged in any or the regular occurrence of disordered eating behaviors (dietary restraint, binge eating distinguished by loss of control) and compensatory behaviors (self‐induced vomiting, use of laxatives, excessive exercising) during the preceding 28 days

	Range	Any occurrence (%), *n* = 382	Regular occurrence (%), *n* = 382
Dietary restraint	—	30.4	10.7
Binge eating distinguished by loss of control	0–96	43.5	18.6
Self‐induced vomiting	0–33	5.2	2.6
Laxative misuse	0–26	4.2	1.6
Excessive exercising	0–40	51.3	7.6

Note

A regular occurrence was determined as ≥4 times during the preceding 28 days. An exclusion to this criterion was applied to dietary restraint (regular occurrence was defined as ≥13 days over the preceding 28 days) and excessive exercise (regular occurrence was defined as ≥20 times over the preceding 28 days). Dietary restraint was a behavior described as going for “long periods of time (>8 hr) without eating anything at all in order to influence your shape or weight” (LT‐EDE‐Q 6.0 item 2); binge eating distinguished by loss of control (or objective binge eating) was an episode described by eating a large amount of food with the feeling of losing self‐control during consumption (LT‐EDE‐Q 6.0 item 14); self‐induced vomiting was an episode described as making “yourself vomit as a means of controlling your shape or weight” (LT‐EDE‐Q 6.0 item 16); laxative misuse was an episode described as going “to take laxatives as a means of controlling your shape or weight” (LT‐EDE‐Q 6.0 item 17); and excessive exercising was an episode described as exercising vigorously in “a driven or compulsive way as a means of controlling your weight, shape or amount of fat, or to burn off calories” (LT‐EDE‐Q 6.0 item 18).

The level of construct validity (divergent validity) and internal consistency of the LT‐EDE‐Q 6.0 are displayed in Table 3. Test‐retest reliability was good to excellent for the general and subscale scores (0.66–0.91) and for the items that assessed essential behavioral features of EDs (0.84–0.90) except for item 14 (episodes of binge eating distinguished by loss of control ICC = 0.41). Cronbach's alpha for each subscale exceeded 0.75 and the LT‐EDE‐Q 6.0 general scale was 0.94. The correlations between the items outside the initial subscale were generally weaker than the interitem correlations. The interitem correlations ranged from 0.37 (eating concern subscale) to 0.50 (shape concern subscale). The correlations between items and subscales other than their own were between 0.31 (restraint subscale) and 0.43 (shape concern subscale).

**Table 3:** Reliability and validity of the LT‐EDE‐Q 6.0

	Test‐retest reliability (ICC)	Cronbach's α	Interitem correlation^a^	Divergent validity^b^
Restraint subscale	0.66	0.83	0.49	0.31
Eating concern subscale	0.84	0.75	0.37	0.33
Shape concern subscale	0.91	0.88	0.50	0.43
Weight concern subscale	0.90	0.83	0.49	0.40
LT‐EDE‐Q 6.0 general scale	0.90	0.94	0.40	
Essential behavioral features of EDs during the preceding 28 days				
Episodes of binge eating (LT‐EDE‐Q 6.0 item 13)	0.90			
Episodes of binge eating distinguished by loss of control (LT‐EDE‐Q 6.0 item 14)	0.41			
Occurrence in days of binge eating distinguished by loss of control (LT‐EDE‐Q 6.0 item 15)	0.86			
Episodes of self‐induced vomiting (LT‐EDE‐Q 6.0 item 16)	—			
Episodes of use of laxatives (LT‐EDE‐Q 6.0 item 17)	—			
Episodes of excessive exercising (LT‐EDE‐Q 6.0 item 18)	0.84			

Note

ICC = intraclass correlation coefficient; for the items 16 and 17 ICC cannot be calculated because of no response variation. LT‐EDE‐Q 6.0 general scale = the combined subscales of the Lithuanian version of Eating Disorder Examination Questionnaire.

a Mean value of Pearson correlations coefficients between items within the assigned subscale.

b Mean value of Pearson correlations coefficients between items in subscales other than their own.

Next, the concurrent validity of the LT‐EDE‐Q 6.0 was assessed by testing the associations with tools of similar constructs (Table 4). The analysis demonstrated these associations in the expected direction between the LT‐EDE‐Q 6.0 scores and the LT‐MBSRQ‐AS, LT‐WHOQOL‐BREF, RSES, and BMI measures. The LT‐EDE‐Q 6.0 general score was moderately and negatively correlated with the LT‐MBSRQ‐AS appearance evaluation and body area satisfaction scores (−0.58, and −0.53, respectively, *p* < .01) but positively correlated with the LT‐MBSRQ‐AS appearance orientation, overweight preoccupation, and self‐classified weight scores (0.27, 0.73, and 0.58, respectively, *p* < .01). The correlation was strong for the LT‐EDE‐Q 6.0 general scores with the LT‐MBSRQ‐AS overweight preoccupation scores. As expected, the LT‐WHOQOL‐BREF scores were negatively associated with the LT‐EDE‐Q 6.0 scores. There were weak to moderate negative correlations between the LT‐WHOQOL‐BREF domain scores and the LT‐EDE‐Q 6.0 subscale scores (*r* = −.11 to −.36, *p* < .01), while the correlation was highest for the LT‐WHOQOL‐BREF psychological domain scores with the LT‐EDE‐Q 6.0 general scores (*r* = −.36, *p* < .01). The RSES scores were negatively associated with the LT‐EDE‐Q 6.0 scores. The correlations between the RSES scores and the restraint, eating concern, shape concern, weight concern, and LT‐EDE‐Q 6.0 general scores were as follows: −0.07, −0.27, −0.25, −0.26, and −0.24, respectively (*p* < .01). In addition, the LT‐EDE‐Q 6.0 general scores were positively associated with the BMI scores (*r* = .36, *p* < .01).

**Table 4:** Correlations between the LT‐EDE‐Q 6.0 scores and LT‐MBSRQ‐AS, LT‐WHOQOL‐BREF, RSES, and BMI measures

	LT‐EDE‐Q 6.0				
	Restraint	Eating concern	Shape concern	Weight concern	LT‐EDE‐Q 6.0 general
LT‐MBSRQ‐AS					
Appearance evaluation	−0.29**	−0.40**	−0.62**	−0.60**	−0.58**
Appearance orientation	0.21**	0.13*	0.29**	0.23**	0.27**
Overweight preoccupation	0.60**	0.54**	0.67**	0.67**	0.73**
Body areas satisfaction	−0.21**	−0.40**	−0.59**	−0.55**	−0.53**
Self‐classified weight	0.38**	0.40**	0.57**	0.57**	0.58**
LT‐WHOQOL‐BREF					
Physical	−0.07	−0.27**	−0.22**	−0.22**	−0.22**
Psychological	−0.13*	−0.32**	−0.40**	−0.35**	−0.36**
Social relationships	0.001	−0.16**	−0.12*	−0.10	−0.11**
Environment	−0.07	−0.22**	−0.15**	−0.15**	−0.16**
RSES	−0.07	−0.27**	−0.25**	−0.26**	−0.24**
BMI	0.23**	0.23**	0.35**	0.39**	0.36**

Abbreviations: BMI, body mass index; LT‐EDE‐Q 6.0, Lithuanian version of the Eating Disorder Examination Questionnaire 6.0; LT‐MBSRQ‐AS, Lithuanian version of the Multidimensional Body‐Self Relations Questionnaire–Appearance Scales; LT‐WHOQOL‐BREF, Lithuanian version of the World Health Organization Quality of Life‐BREF Questionnaire; RSES, M. Rosenberg Self‐Esteem Scale.

* *p* < .05.

** *p* < .01.

Finally, CFA of the 22 attitudinal items of the LT‐EDE‐Q 6.0 was carried out to verify the consistency of its factorial structure with that of the original version. The original 4‐factor structure was not obtained by CFA. After conducting CFA, we found poor model goodness of fit for the original 4‐factor structure of the EDE‐Q 6.0 in the present mixed‐gender nonclinical student sample (*χ* ^2^/*df* = 9.730, *p* < .0001; GFI = 0.660; CFI = 0.710; AGFI = 0.576; TLI = 0.670; RMSEA = 0.151). Then, a series of different proposed models were run, but in the Lithuanian sample, none of them was confirmed (Appendix 1). Invariance analyses across gender groups revealed a statistical difference between unconstrained and fully constrained models (Appendix 2). The statistically significant difference was found when testing the assumption about factors loadings and measurement residuals equality across genders (*p* < .001) but not structural covariances (*p* = .233).

## 4 DISCUSSION

In the present study, we aimed to introduce a Lithuanian version of the EDE‐Q 6.0 as a screening self‐report questionnaire for EDs in a nonclinical Lithuanian student sample and to verify its reliability, validity, and factor structure with different psychometric tests. In general, the results of the current study preliminarily support the applicability, validity, and reliability of the LT‐EDE‐Q 6.0 in the nonclinical Lithuanian samples.

We found that the LT‐EDE‐Q general mean score of 1.5 ± 1.02 was close to the level of the mixed‐gender sample in the UK (1.63 ± 1.25; Carey et al., 2019). The general mean score for women was higher than in men, and these findings go in line with other studies (Carey et al., 2019; Isomaa et al., 2016; Mitsui et al., 2017; Reas, Øverås, & Rø, 2012; Yucel et al., 2011). For women, general score (1.64 ± 1.22) was higher compared with the female samples of comparable age in Norway (1.42 ± 1.07), Portugal (1.49 ± 1.50) and Spain (1.41 ± 1.19; Rø, Reas, & Lask, 2010). However, the general score for women was lower to the samples of US and UK women, accordingly (1.74 ± 1.3 and 1.75 ± 1.25; Carey et al., 2019; Mond, Hay, Rodgers, & Owen, 2006). For men, the general mean score 1.08 ± 1.07 was higher than in the student sample from Norway (0.44 ± 0.52; Reas et al., 2012) and Spain (0.58 ± 0.83; Peláez‐Fernández, Labrador, & Raich, 2013) but close to UK and US men, accordingly (1.16 ± 1.11 and 1.09 ± 1.00; Carey et al., 2019; Lavender, De Young, & Anderson, 2010).

There were found some similarities between the Lithuanian and Spanish students regarding the episodes of regular binge eating distinguished by loss of control. Approximately 19% of the study participants engaged in regular binge eating distinguished by loss of control, compared with 20.1% in a Spanish sample of undergraduate women (Villarroel, Penelo, Portell, & Raich, 2011). Similar results were found between the Lithuanian students and Portuguese college women on the episodes of regular self‐induced vomiting (Machado et al., 2014) and between our Lithuanian study sample and Norwegian university women on the episodes of excessive exercising (Rø et al., 2010). The frequency of the regular occurrence of dietary restraint in our sample was approximately 11%, which appears to be much higher than the 1.8% in the Norway study (Rø et al., 2010), but lower than the 17.6% in the Portugal study (Machado et al., 2014). Our study findings indicated that laxative misuse is a common phenomenon in our student sample.

We found high Cronbach's alpha coefficients for the general and four subscales of the LT‐EDE‐Q 6.0 (ranging from 0.75 to 0.94) that were in line with those seen in other studies in nonclinical student samples (Giovazolias et al., 2013; Rø et al., 2010; Villarroel et al., 2011; Yucel et al., 2011), and these results indicated an acceptable to excellent level of internal consistency for our Lithuanian version. Moreover, the test‐retest reliability of the LT‐EDE‐Q 6.0 general and subscales was good to excellent (ICC range was 0.66–0.91), as it was for the items that assessed the essential behavioral features of EDs (ICC range was 0.84–0.90, with the exception of item 14, ICC = 0.4). Similar results were found in a Norwegian female university student sample, where test‐retest reliability, evaluated by Spearman rank correlations, for the global EDE‐Q and subscale scores were high (0.82–0.93), and this reliability was slightly lower for the occurrence and frequency of ED behaviors (0.71–0.83; Rø et al., 2010).

The Lithuanian version of the questionnaire demonstrated adequate concurrent validity. The pattern of correlations between the LT‐EDE‐Q 6.0 general and subscale scores and the LT‐MBSRQ‐AS subscale scores confirmed our beliefs of the connection between ED psychopathology and several body‐related concepts. First, the appearance evaluation and body area satisfaction subscales were significantly and negatively associated with the LT‐EDE‐Q 6.0 general and subscale scores. As expected, one's satisfaction and enjoyment with one's physical appearance or specific areas of the body were negatively associated with one's weight, shape, and eating concerns as well as with food restraint measured by the LT‐EDE‐Q 6.0. These findings are in line with the Hebrew validation study, where another instrument that assessed a similar concept (the Dresden Body Image Questionnaire‐35, DKB‐35) was used to analyze the connection between eating symptomatology and several body‐related concepts (Zohar et al., 2017). Second, our study showed that the appearance orientation, overweight preoccupation, and self‐classified weight measures had significant and positive associations with the LT‐EDE‐Q 6.0 general and subscale scores. Our results might support the fact that young people who are preoccupied with their weight and physical attractiveness, in general, are more engaged in EDs and health‐compromising eating behaviors. Other validation studies have also shown that there are positive and significant correlations between EDE‐Q scores and other measures indicating body dissatisfaction (Giovazolias et al., 2013; Yucel et al., 2011; Zohar et al., 2017).

The LT‐EDE‐Q 6.0 general and subscale scores had negative and weak to moderate correlations with the scores from the LT‐WHOQOL‐BREF and its domains (−0.11 to −0.36). These results partially follow the previous study that indicated a significant and negative association between the EDE‐Q and another tool that evaluated a related concept—the satisfaction with life scale (SWLS; −0.10 to −0.34; Zohar et al., 2017). In our study, the correlation was highest for the LT‐WHOQOL‐BREF psychological domain scores with the LT‐EDE‐Q 6.0 general scores and provided further evidence for findings reported in a literature review of clinical psychology demonstrating that patients with EDs have a poor quality of life, especially in the psychosocial domain (Jenkins, Hoste, Meyer, & Blissett, 2011).

As self‐esteem is an important etiological factor in EDs (Jacobi, Hütter, & Fittig, 2018), we found a negative relationship between the RSES and LT‐EDE‐Q 6.0 general scores, and these results were also in line with other findings (Mitsui et al., 2017).

We found a positive and significant correlation between the LT‐EDE‐Q 6.0 scores and BMI (0.36, *p* < .01). These findings coincide with other studies conducted in a sample of adolescents and adults in Finland (0.28 and 0.40, respectively; Isomaa et al., 2016), in a sample of community volunteers in Israel (0.34; Zohar et al., 2017), and in a sample of Turkish primary and high school students (0.36; Yucel et al., 2011). This fact might add to the knowledge that young people seeking to lose bodyweight frequently use maladaptive strategies for controlling their weight.

Finally, we expected that the factor structure of the LT‐EDE‐Q 6.0 in a nonclinical Lithuanian student sample would reflect the original 4‐factor structure. This assumption was not confirmed, although the findings were consistent with other studies evaluating the factor structure of the EDE‐Q 6.0 in a student sample (Giovazolias et al., 2013; Grilo et al., 2015; Machado et al., 2018; Tobin et al., 2019). In the present study, our CFA findings supported a poor model goodness of fit for the original version of the questionnaire. Unfortunately, further testing of different other proposed models (Fairburn & Beglin, 1994; Giovazolias et al., 2013; Peterson et al., 2007) did not confirm the expected results. In agreement with a study conducted by Calugi et al. (2017), these findings might be explained by the fact that the initial EDE and EDE‐Q subscales were deliberately developed to include items collected together based on a representation of significant areas of ED psychopathology (Cooper, Cooper, & Fairburn, 1989) rather than on factor analysis. Therefore, assessing the general scale score without application of the subscales is recommended in student samples. However, future studies should continue testing LT‐EDE‐Q 6.0 in other samples of men and women.

The present study has some important limitations worth mentioning. The majority of our sample was female. Studies have demonstrated that university and college students, especially females, have been reported to present with high rates of ED symptoms (Eisenberg, Nicklett, Roeder, & Kirz, 2011; Keski‐Rahkonen & Mustelin, 2016). Therefore, future studies should test LT‐EDE‐Q 6.0 with an equal distribution of women and men and samples of various ages. Further, the direct cross‐cultural comparisons of the normative results are limited due to the country's cultural differences and methodological differences. Additionally, the present sample does not represent the community of Lithuanian students. Next, since the EDE‐Q is a clinical tool, it should be used with clinical samples. Further psychometric studies involving a more clinically based sample to identify clinically diagnosed cases are needed.

## 5 CONCLUSIONS

In general, the results of the current study preliminarily support the applicability, validity, and reliability of the LT‐EDE‐Q 6.0 in a nonclinical Lithuanian student sample. However, we recommend assessing the general scale score without the application of the subscales. The Lithuanian version of this instrument should be further investigated with more diverse and more extensive populations, involving gender differences, more comprehensive age ranges, and various clinical samples to identify clinically diagnosed cases.

## CONFLICTS OF INTEREST

The authors declare no conflict of interest.

## AUTHOR CONTRIBUTIONS

RJ and MB involved in conceptualization; RJ, MB, and VB involved in methodology, validation, investigation, resources, writing original draft preparation, and writing‐review and editing; MB, and VB involved in software, formal analysis, and data curation; RJ involved in supervision and project administration.

## Data Availability

The dataset generated and analyzed during the current study is not publicly available but is available from the corresponding author on reasonable request.

## Appendix 1

EDE‐Q confirmatory factor analysis

**Table:**

Model	CMIN/DF	*p*	GFI	AGFI	TLI	CFI	RMSEA
Original (Fairburn and Beglin, 1994)	9.730	<.0001	0.660	0.576	0.670	0.710	0.151
Three factors (Peterson et al., 2007)	9.796	<.0001	0.647	0.566	0.668	0.704	0.152
One factor (Giovazolias et al., 2013)	12.315	<.0001	0.554	0.460	0.573	0.613	0.172

## Appendix 2

EDE‐Q confirmatory factor analysis: structural invariance analysis across genders

**Table:**

Models	CMIN/DF	*p*	GFI	AGFI	TLI	CFI	RMSEA
Unconstrained model (general fit across genders)	5.782	<.0001	0.636	0.546	0.645	0.688	0.112
Men (*n* = 95)	3.539	<.0001	0.611	0.516	0.652	0.694	0.164
Women (*n* = 287)	8.021	<.0001	0.645	0.557	0.643	0.686	0.157
Constrained models							
Measurement weights	5.677	<.0001	0.626	0.553	0.653	0.682	0.111
Structural covariances	5.576	<.0001	0.622	0.560	0.661	0.681	0.110
Measurement residuals	5.637	<.0001	0.612	0.569	0.656	0.661	0.110

## References

[brb31555-bib-0001] Aputytė, V. (2000). Nervinės anoreksijos ir nervinės bulimijo klinikiniai psichologiniai ypatumai Lietuvoje: Biomedicinos mokslų daktaro disertacijos santrauka (The clinical psychologycal aspects of nervous anorexia and bulimia in Lithuania: The summary of the doctoral dissertation).

[brb31555-bib-0002] Baceviciene, M. , Jankauskiene, R. , & Emeljanovas, A. (2019). Self‐perception of physical activity and fitness is related to lower psychosomatic health symptoms in adolescents with unhealthy lifestyles. BMC Public Health, 19(1), 980 10.1186/s12889-019-7311-2 31337374 PMC6647301

[brb31555-bib-0003] Berg, K. C. , Peterson, C. B. , Frazier, P. , & Crow, S. J. (2012). Psychometric evaluation of the eating disorder examination and eating disorder examination‐questionnaire: A systematic review of the literature. International Journal of Eating Disorders, 45(3), 428–438. 10.1002/eat.20931 21744375 PMC3668855

[brb31555-bib-0004] Brown, T. A. , Cash, T. F. , & Mikulka, P. J. (1990). Attitudinal body‐image assessment: Factor analysis of the body‐self relations questionnaire. Journal of Personality Assessment, 55(1–2), 135–144. 10.1207/s15327752jpa5501&2_13 2231236

[brb31555-bib-0005] Byrne, S. M. , Allen, K. L. , Lampard, A. M. , Dove, E. R. , & Fursland, A. (2010). The factor structure of the eating disorder examination in clinical and community samples. International Journal of Eating Disorders, 43(3), 260–265. 19350647 10.1002/eat.20681

[brb31555-bib-0006] Calugi, S. , Milanese, C. , Sartirana, M. , El Ghoch, M. , Sartori, F. , Geccherle, E. , … Dalle Grave, R. (2017). The Eating Disorder Examination Questionnaire: Reliability and validity of the Italian version. Eating and Weight Disorders‐Studies on Anorexia, Bulimia and Obesity, 22(3), 509–514. 10.1007/s40519-016-0276-6 27039107

[brb31555-bib-0007] Carey, M. , Kupeli, N. , Knight, R. , Troop, N. A. , Jenkinson, P. M. , & Preston, C. (2019). Eating disorder examination questionnaire (EDE‐Q): Norms and psychometric properties in UK females and males. Psychological Assessment, 31(7), 839–850. 30802119 10.1037/pas0000703

[brb31555-bib-0008] Ciao, A. , Loth, K. , & Neumark‐Sztainer, D. (2014). Preventing eating disorder pathology: Common and unique features of successful eating disorders prevention programs. Current Psychiatry Reports, 16(7), 1–13. 10.1007/s11920-014-0453-0 PMC4104660 24821099

[brb31555-bib-0009] Cooper, Z. , Cooper, P. J. , & Fairburn, C. G. (1989). The validity of the eating disorder examination and its subscales. The British Journal of Psychiatry, 154(6), 807–812. 10.1192/bjp.154.6.807 2597887

[brb31555-bib-0010] Cooper, Z. , & Fairburn, C. (1987). The eating disorder examination: A semi‐structured interview for the assessment of the specific psychopathology of eating disorders. International Journal of Eating Disorders, 6(1), 1–8. 10.1002/1098-108X(198701)6:1<1:AID-EAT2260060102>3.0.CO;2-9

[brb31555-bib-0011] Dučinskienė, D. , Kalėdinė, R. , Petrauskienė, J. , & Šumskas, L. (2002). Pasaulio sveikatos organizacijos klausimyno tinkamumo įvertinimas studentų gyvenimo kokybei tirti. Sveikatos Mokslai, 3, 53–58.

[brb31555-bib-0012] Eisenberg, D. , Nicklett, E. J. , Roeder, K. , & Kirz, N. E. (2011). Eating disorder symptoms among college students: Prevalence, persistence, correlates, and treatment‐seeking. Journal of American College Health, 59(8), 700–707. 10.1080/07448481.2010.546461 21950250 PMC3721327

[brb31555-bib-0013] Fairburn, C. G. , & Beglin, S. J. (1994). Assessment of eating disorders: Interview or self‐report questionnaire? International Journal of Eating Disorders, 16(4), 363–370. 7866415

[brb31555-bib-0014] Fairburn, C. G. , & Beglin, S. J. (2008). Eating disorder examination questionnaire (EDE‐Q 6.0) In Fairburn C. G. (Ed.), Cognitive behavior therapy and eating disorders (pp. 309–313). New York, NY: Guilford Press.

[brb31555-bib-0015] Fairburn, C. G. , & Cooper, Z. (1993). The eating disorder examination In Fairburn C. G., & Wilson G. T. (Eds.), Binge eating: Nature, assessment, and treatment (12th ed., pp. 317–360). New York, NY: Guilford Press.

[brb31555-bib-0016] Fairburn, C. G. , Cooper, Z. , & O'Connor, M. (2008). Eating disorder examination (16.0 D) In Fairburn C. G. (Ed.), Cognitive behavior therapy and eating disorders (pp. 265–308). New York, NY: Guilford Press.

[brb31555-bib-0017] Garner, D. M. (1991). Eating disorder Inventory–2 professional manual. Odessa, FL: Psychological Assessment Resources.

[brb31555-bib-0018] Gideon, N. , Hawkes, N. , Mond, J. , Saunders, R. , Tchanturia, K. , & Serpell, L. (2016). Development and psychometric validation of the EDE‐QS, a 12 item short form of the Eating Disorder Examination Questionnaire (EDE‐Q). PLoS ONE, 11(5), e0152744 10.1371/journal.pone.0152744 27138364 PMC4854480

[brb31555-bib-0019] Giovazolias, T. , Tsaousis, I. , & Vallianatou, C. (2013). The factor structure and psychometric properties of the Greek version of the eating disorders examination questionnaire (EDE‐Q). European Journal of Psychological Assessment, 29(3), 189–196. 10.1027/1015-5759/a000138

[brb31555-bib-0020] Grilo, C. M. , Reas, D. L. , Hopwood, C. J. , & Crosby, R. D. (2015). Factor structure and construct validity of the eating disorder examination‐questionnaire in college students: Further support for a modified brief version. International Journal of Eating Disorders, 48(3), 284–289. 10.1002/eat.22358 25346071 PMC4374034

[brb31555-bib-0021] Hilbert, A. , Tuschen‐Caffier, B. , Karwautz, A. , Niederhofer, H. , & Munsch, S. (2007). Eating disorder examination‐questionnaire. Diagnostica, 53(3), 144–154. 10.1026/0012-1924.53.3.144

[brb31555-bib-0022] Isomaa, R. , Lukkarila, I. , Ollila, T. , Nenonen, H. , Charpentier, P. , Sinikallio, S. , & Karhunen, L. (2016). Development and preliminary validation of a Finnish version of the Eating Disorder Examination Questionnaire (EDE‐Q). Nordic Journal of Psychiatry, 70(7), 542–546. 10.1080/08039488.2016.1179340 27152496

[brb31555-bib-0023] Jacobi, C. , Hütter, K. , & Fittig, E. (2018). Psychosocial risk factors for eating disorders In Agras W. S. & Robinson A. (Eds.), The Oxford handbook of eating disorders (pp. 106–154). New York, NY: Oxford University Press.

[brb31555-bib-0024] Jankauskiene, R. , & Baceviciene, M. (2019). Body image concerns and body weight overestimation do not promote healthy behaviour: Evidence from adolescents in Lithuania. International Journal of Environmental Research and Public Health, 16(5), 864 10.3390/ijerph16050864 PMC6427769 30857295

[brb31555-bib-0025] Jenkins, P. E. , Hoste, R. R. , Meyer, C. , & Blissett, J. M. (2011). Eating disorders and quality of life: A review of the literature. Clinical Psychology Review, 31(1), 113–121. 10.1016/j.cpr.2010.08.003 20817335

[brb31555-bib-0026] Keski‐Rahkonen, A. , & Mustelin, L. (2016). Epidemiology of eating disorders in Europe: Prevalence, incidence, comorbidity, course, consequences, and risk factors. Current Opinion in Psychiatry, 29(6), 340–345. 10.1097/YCO.0000000000000278 27662598

[brb31555-bib-0027] Lavender, J. M. , De Young, K. P. , & Anderson, D. A. (2010). Eating Disorder Examination Questionnaire (EDE‐Q): Norms for undergraduate men. Eating Behaviors, 11(2), 119–121. 10.1016/j.eatbeh.2009.09.005 20188296

[brb31555-bib-0028] Lesinskiene, S. , Girdzijauskiene, S. , Gintiliene, G. , Butkiene, D. , Puras, D. , Goodman, R. , & Heiervang, E. (2018). Epidemiological study of child and adolescent psychiatric disorders in Lithuania. BMC Public Health, 18(1), 548 10.1186/s12889-018-5436-3 29699524 PMC5921298

[brb31555-bib-0029] Machado, P. P. , Grilo, C. M. , & Crosby, R. D. (2018). Replication of a modified factor structure for the eating disorder Examination‐Questionnaire: Extension to clinical eating disorder and non‐clinical samples in Portugal. European Eating Disorders Review, 26(1), 75–80. 10.1002/erv.2569 29152813

[brb31555-bib-0030] Machado, P. P. , Martins, C. , Vaz, A. R. , Conceição, E. , Bastos, A. P. , & Gonçalves, S. (2014). Eating Disorder Examination Questionnaire: Psychometric properties and norms for the Portuguese population. European Eating Disorders Review, 22(6), 448–453. 10.1002/erv.2318 25175299

[brb31555-bib-0031] Mahmoodi, M. , Moloodi, R. , & Ghaderi, A. (2016). The Persian version of Eating Disorder Examination Questionnaire and clinical impairment assessment: Norms and psychometric properties for undergraduate women. Iranian Journal of Psychiatry, 11(2), 67. 27437002 PMC4947222

[brb31555-bib-0032] Miškinytė, A. , & Bagdonas, A. (2010). Jaunų suaugusiųjų požiūrio į savo kūną sąsajos su demografiniais rodikliais. Psichologija, 42, 85–100. 10.15388/Psichol.2010.0.2569

[brb31555-bib-0033] Mitsui, T. , Yoshida, T. , & Komaki, G. (2017). Psychometric properties of the eating disorder examination‐questionnaire in Japanese adolescents. BioPsychoSocial Medicine, 11(1), 9 10.1186/s13030-017-0094-8 28392830 PMC5379552

[brb31555-bib-0034] Mond, J. M. , Hay, P. J. , Rodgers, B. , & Owen, C. (2006). Eating Disorder Examination Questionnaire (EDE‐Q): Norms for young adult women. Behaviour Research and Therapy, 44(1), 53–62. 10.1016/j.brat.2004.12.003 16301014

[brb31555-bib-0035] Neumark‐Sztainer, D. , Wall, M. M. , Chen, C. , Larson, N. I. , Christoph, M. J. , & Sherwood, N. E. (2018). Eating, activity, and weight‐related problems from adolescence to adulthood. American Journal of Preventive Medicine, 55(2), 133–141. 10.1016/j.amepre.2018.04.032 29937114 PMC6072273

[brb31555-bib-0036] Peláez‐Fernández, M. A. , Labrador, F. J. , & Raich, R. M. (2012). Validation of Eating Disorder Examination Questionnaire (EDE‐Q)–Spanish version–for screening eating disorders. The Spanish Journal of Psychology, 15(2), 817–824. 10.5209/rev_SJOP.2012.v15.n2.38893 22774455

[brb31555-bib-0037] Peláez‐Fernández, M. A. , Labrador, F. J. , & Raich, R. M. (2013). Norms for the Spanish version of the eating disorders examination questionnaire (S‐EDE‐Q). Psicothema, 25(1), 107–114. 23336552 10.7334/psicothema2012.18

[brb31555-bib-0038] Peterson, C. B. , Crosby, R. D. , Wonderlich, S. A. , Joiner, T. , Crow, S. J. , Mitchell, J. E. , … le Grange, D. (2007). Psychometric properties of the eating disorder examination‐questionnaire: Factor structure and internal consistency. International Journal of Eating Disorders, 40(4), 386–389. 10.1002/eat.20373 17304585

[brb31555-bib-0039] Reas, D. L. , Øverås, M. , & Rø, Ø. (2012). Norms for the Eating Disorder Examination Questionnaire (EDE‐Q) among high school and university men. Eating Disorders, 20(5), 437–443. 10.1080/10640266.2012.715523 22985240

[brb31555-bib-0040] Rø, Ø. , Reas, D. L. , & Lask, B. (2010). Norms for the Eating Disorder Examination Questionnaire among female university students in Norway. Nordic Journal of Psychiatry, 64(6), 428–432. 10.3109/08039481003797235 20429744

[brb31555-bib-0041] Rosenberg, M. (1979). Conceiving the self. New York, NY: Basic Books.

[brb31555-bib-0042] Schmitt, D. P. , & Allik, J. (2005). Simultaneous administration of the Rosenberg self‐esteem scale in 53 nations: Exploring the universal and culture‐specific features of global self‐esteem. Journal of Personality and Social Psychology, 89(4), 623 10.1037/0022-3514.89.4.623 16287423

[brb31555-bib-0043] Tobin, L. N. , Lacroix, E. , & von Ranson, K. M. (2019). Evaluating an abbreviated three‐factor version of the Eating Disorder Examination Questionnaire in three samples. Eating Behaviors, 32, 18–22. 10.1016/j.eatbeh.2018.11.003 30476704

[brb31555-bib-0044] Unikel Santoncini, C. , Bojorquez Chapela, I. , de León, D. , Vázquez, C. , Vázquez Velázquez, V. , Rivera Márquez, J. A. , … Rocha Velis, I. (2018). Validation of eating disorders examination questionnaire in Mexican women. International Journal of Eating Disorders, 51(2), 146–154. 10.1002/eat.22819 29314174

[brb31555-bib-0045] Villarroel, A. M. , Penelo, E. , Portell, M. , & Raich, R. M. (2011). Screening for eating disorders in undergraduate women: Norms and validity of the Spanish version of the Eating Disorder Examination Questionnaire (EDE‐Q). Journal of Psychopathology and Behavioral Assessment, 33(1), 121–128. 10.1007/s10862-009-9177-6

[brb31555-bib-0046] Wade, T. D. , Byrne, S. , & Bryant‐Waugh, R. (2008). The eating disorder examination: Norms and construct validity with young and middle adolescent girls. International Journal of Eating Disorders, 41(6), 551–558. 10.1002/eat.20526 18433026

[brb31555-bib-0047] World Health Organization (1997). Obesity: Preventing and managing the global epidemic: Report of a WHO consultation on obesity, Geneva, 3–5 June 1997 (No. WHO/NUT/NCD/98.1). Geneva, Switzerland: World Health Organization. 11234459

[brb31555-bib-0048] World Health Organization (1998a). Development of the world health organization WHOQOL‐BREF quality of life assessment. Psychological Medicine, 28(3), 551–558. 9626712 10.1017/s0033291798006667

[brb31555-bib-0049] World Health Organization (1998b). Programme on mental health: WHOQOL user manual. Geneva, Switzerland: World Health Organization.

[brb31555-bib-0050] Yucel, B. , Polat, A. , Ikiz, T. , Dusgor, B. P. , Elif Yavuz, A. , & Sertel Berk, O. (2011). The Turkish version of the Eating Disorder Examination Questionnaire: Reliability and validity in adolescents. European Eating Disorders Review, 19(6), 509–511. 10.1002/erv.1104 21400637

[brb31555-bib-0051] Zohar, A. H. , Lev‐Ari, L. , & Bachner‐Melman, R. (2017). The EDE‐Q in Hebrew: Structural and convergent/divergent validity in a population sample. The Israel Journal of Psychiatry and Related Sciences, 54(3), 15–20. 29735808

